# Barriers and factors associated with patient-centered communication in oncology inpatients: a cross-sectional study

**DOI:** 10.1590/1980-220X-REEUSP-2025-0265en

**Published:** 2026-11-06

**Authors:** Betülay Kiliç, Serap Korkmaz, Selcan Erel Kurt, Fatma İlknur Çinar

**Affiliations:** 1 University of Health Sciences, Gulhane Faculty of Nursing, Ankara, Türkiye.; 2 Cyprus International University, Faculty of Health Sciences, Lefkoşa, Türkiye.

**Keywords:** Communication Barriers, Nurse-Patient Relations, Oncology Nursing, Patient-Centered Care, Barreiras de Comunicação, Relações Enfermeiro-Paciente, Enfermagem Oncológica, Assistência Centrada no Paciente

## Abstract

**Objective:**

This study aimed to evaluate patient-centered communication barriers in oncology patients and the factors influencing these barriers.

**Methods:**

A cross-sectional study was conducted with 336 hospitalized cancer patients (≥18 years) in the Oncology Clinic of a Training and Research Hospital between February 2022 and March 2023. Patients with dementia or intellectual disability were excluded. Data were collected through face-to-face interviews using a Sociodemographic Form and the Patient-Centered Communication Barriers Scale (PCCBS).

**Results:**

The mean age of participants was 57.5 ± 17.3 years; 68.7% were male, and the mean hospitalization period was 13.4 ± 15.9 days. Regression analysis showed significant relationships between patient characteristics and communication barriers. Higher education increased patients’ need for attention and information (β = 0.175, p = 0.001, 95% CI [0.039–0.157]) and curiosity (β = 0.130, p = 0.017, 95% CI [0.024–0.242]). Greater symptom burden reduced the need for attention (β = –0.116, p = 0.031, 95% CI [–0.345, –0.016]). Absence of a caregiver was associated with higher fear and anxiety (β = 0.173, p = 0.001, 95% CI [0.180–0.749]). Education and symptom experience explained 4.5% of the variance in need for attention (R2 = 0.045), 1.7% in curiosity and information needs (R2 = 0.017), and 3% in fear and anxiety (R2 = 0.030).

**Conclusion:**

Patients with higher education reported greater information needs, whereas those with heavier symptom burdens were less likely to seek attention. Lack of caregiver support increased fear and anxiety. These results indicate that communication barriers in oncology are influenced by educational background, symptom experience, and caregiving context. Nurses and clinicians should assess these factors systematically and tailor communication strategies to individual needs. Implementing such interventions and screening for barriers can enhance patient-centered oncology care. However, the cross-sectional, single-center design limits causal inference and generalizability.

## RESUMO

**Objetivo:**

Este estudo teve como objetivo avaliar as barreiras de comunicação centradas no paciente em pacientes oncológicos e os fatores que influenciam essas barreiras.

**Métodos:**

Foi realizado um estudo transversal com 336 pacientes oncológicos hospitalizados (≥18 anos) na Clínica de Oncologia de um Hospital de Ensino e Pesquisa entre fevereiro de 2022 e março de 2023. Pacientes com demência ou deficiência intelectual foram excluídos. Os dados foram coletados por meio de entrevistas presenciais utilizando um Formulário Sociodemográfico e a Escala de Barreiras de Comunicação Centradas no Paciente (PCCBS).

**Resultados:**

A idade média dos participantes foi de 57,5 ± 17,3 anos; 68,7% eram do sexo masculino e o período médio de internação foi de 13,4 ± 15,9 dias. A análise de regressão mostrou relações significativas entre as características dos pacientes e as barreiras de comunicação. Níveis mais elevados de escolaridade aumentaram a necessidade de atenção e informação dos pacientes (β = 0,175, p = 0,001, IC 95% [0,039-0,157]) e a curiosidade (β = 0,130, p = 0,017, IC 95% [0,024–0,242]). Uma maior carga de sintomas reduziu a necessidade de atenção (β = –0,116, p = 0,031, IC 95% [–0,345, –0,016]). A ausência de um cuidador foi associada a maior medo e ansiedade (β = 0,173, p = 0,001, IC 95% [0,180–0,749]). A educação e a experiência com os sintomas explicaram 4,5% da variância na necessidade de atenção (R2 = 0,045), 1,7% na curiosidade e nas necessidades de informação (R2 = 0,017) e 3% no medo e na ansiedade (R2 = 0,030).

**Conclusão:**

Pacientes com maior escolaridade relataram maiores necessidades de informação, enquanto aqueles com maior carga de sintomas apresentaram menor probabilidade de buscar atenção. A falta de apoio do cuidador aumentou o medo e a ansiedade. Esses resultados indicam que as barreiras de comunicação em oncologia são influenciadas pela formação educacional, pela experiência com os sintomas e pelo contexto de cuidado. Enfermeiros e clínicos devem avaliar esses fatores sistematicamente e adaptar as estratégias de comunicação às necessidades individuais. A implementação dessas intervenções e a triagem para identificar barreiras podem aprimorar o cuidado oncológico centrado no paciente. No entanto, o desenho transversal e unicêntrico limita a inferência causal e a generalização dos resultados.

## INTRODUCTION

Cancer is a devastating health problem that leads to different experiences depending on patients’ responses to treatment throughout the diagnosis and treatment process. It remains one of the leading causes of morbidity and mortality worldwide, with approximately 20 million new cases and 9.7 million deaths reported annually. These figures illustrate the global burden of cancer and underscore the importance of effective communication in oncology care to address patients’ psychological and informational needs^(1)^. Communication in cancer care is crucial due to the sensitive nature of the information and its profound psychosocial impact on patients and their families^(2)^. Effective communication can motivate disease prevention, behavior change, and health promotion^(3)^. Beyond the treatment process, one of the essential roles of the healthcare team is to ensure that the messages conveyed are consistent with the patient’s individual needs and preferences^(4)^.

Patient-centered communication can directly and indirectly contribute to identifying patients’ needs and concerns, as well as understanding, satisfaction, motivation, trust, and overall health^(5,6)^. This approach is crucial to delivering high-quality cancer care and can be effective in various ways, including health promotion, protection, and disease prevention. Effective health communication can help patients and their families make informed decisions, cope with cancer, and manage the care process effectively^(7)^. Inadequate communication, on the other hand, can lead to care interruptions, treatment refusal, and various mistakes^(6)^. It also enhances the quality of healthcare and contributes to taking precautions by increasing individuals’ awareness of the risk of disease^(5,8)^.

Understanding and empathizing with patients’ perspectives on the disease are critical components of patient-centered communication^(9)^. This communication approach reveals patients’ concerns, ideas, expectations, needs, emotions, and unique psychosocial and cultural environment. Additionally, the patient and healthcare team can establish a shared understanding of treatments that align with the patient’s values and preferences^(10)^. Effective communication between patients and providers increases patients’ participation in health services and has a positive impact on service quality and health outcomes^(11)^.

Patient-centered communication requires nurses to develop a shared understanding of the problem, treatment goals, and barriers to health. This approach involves accepting the person’s personality, life history, and social context^(12)^. Patient-centered communication refers to the processes and outcomes of interactions between nurses, service providers, and patients. The quality of health services depends on the relationship between the patient and the health worker. Nurses, in particular, play a critical role in patient-centered care, and the success of this care also depends on their effective communication with patients ^(13)^. Patient-centered communication has been shown to prevent patients from withdrawing from treatment and to yield better clinical outcomes^(14,15)^. In the existing literature, factors such as patient education, lack of information, symptom burden, and caregiver support are associated with communication barriers^(16,17,18)^. Moreover, some studies have revealed that patient and family factors are considered the most significant barriers to discussions about care goals^(19)^. At the same time, recent frameworks, such as the Patient-Centered Communication Barriers Scale (PCCBS), highlight that communication barriers occur across multiple domains of the patient–provider interaction^(20)^. Each domain reflects a distinct challenge to implementing the principles of patient- centered care.

For example, the lack of perceived attention may reinforce patients’ feelings of being undervalued, thereby undermining trust and open communication^(21)^. Fear and anxiety may prevent patients from asking questions or actively participating in the treatment process, positioning them in a passive role. Similarly, unmet curiosity and information needs hinder patients’ understanding of their condition and treatment, creating uncertainty, misinterpretations, and communication gaps^(22)^. Environmental and organizational factors also play a significant role; long waiting times may lead to impatience, fatigue, and frustration, while negative encounters with security staff can diminish patients’ sense of comfort and safety, ultimately impairing communication^(23)^. Finally, prejudice and mistrust can hinder the establishment of a therapeutic relationship, weakening collaboration in treatment^(24)^. Among the barriers faced by oncology nurses in patient-centered communication, the lack of time, deficiencies in team communication, and limited empathy skills are particularly prominent^(5)^. However, the scope and context of these barriers among hospitalized oncology patients have not yet been sufficiently investigated. This study aims to systematically evaluate patient-centered communication barriers and related factors in hospitalized oncology inpatients, thereby contributing to the improvement of clinical practice.

## METHODS

### Study Design and Sample

This cross-sectional study was conducted with 336 cancer patients hospitalized in the Oncology Clinic of a Training and Research Hospital between February 2022 and March 2023. The total number of patients who applied to the Oncology Clinic was 2990, including 524 repeat admissions. To avoid counting the same patients multiple times, an adequate population size was considered as 2466. The sample size was calculated using the formula established by Salant and Dillman (1994):


$$n=\frac{Nt^{2}pq}{d^{2}\left(N-1\right)+t^{2}pq}$$

N: Total population size.

n: Number of individuals to be sampled.

p: Frequency of occurrence of the studied event (probability of occurrence).

q: Frequency of non-occurrence of the studied event (probability of non-occurrence).

t: Theoretical value from the t-table corresponding to a given significance level.

d: Accepted margin of error for the occurrence frequency of the event.

The sample size was calculated using Salant and Dillman’s formula for this non-homogeneous population with a 95% confidence interval and ± 5% sampling error, and the required sample size was n = 2466 (1.96)2 (0.50) (0.50)/ (0.05)2 (2466 – 1) + (1.96)2 (0.50) (0.50) = 332 participants^(25)^. Participants were recruited using a consecutive sampling approach. During the data collection period, all inpatients who met the inclusion criteria and agreed to participate were consecutively invited to the study. Individuals aged 18 years and over, diagnosed with cancer, and who agreed to participate were included. Patients with a known diagnosis of dementia or intellectual disability were excluded at screening. Data were collected from 340 patients; however, 4 patients were excluded from the analysis due to missing data, yielding a final sample of 336 participants. The Strengthening the Reporting of Observational Studies in Epidemiology (STROBE) guidelines were used as a guide for methodological rigor^(26)^.

#### Participants

- Inpatients in oncology unit & Outpatients receiving chemotherapy (n = 340).
- Excluded: Missing or incomplete data (n = 4).
- Final sample included in analysis (n = 336).

### Data Collection

Data were collected using the “Sociodemographic Form” and “Patient-Centered Communication Barriers Scale (PCCBS)”. The researchers collected data using the face-to-face interview method. The patients were asked to sign an informed consent form before data collection. Data collection took approximately 7-8 minutes for each patient.

#### The Sociodemographic Form

The sociodemographic form completed by the study participants consisted of 18 questions. It included questions on year of birth, gender, marital status, income level, number of children, educational status, type of cancer, presence of comorbidities, duration of hospitalization, and symptom experience.

#### Patient-Centered Communication Barriers Scale (PCCBS)

The patient-centered communication barriers scale comprises 28 questions organized into seven subgroups: need for attention, security personnel, fear and anxiety, curiosity and need for information, waiting time, prejudice, and distrust. The scale comprises 6 questions about the need for attention, 4 questions about security personnel, 4 questions about fear and anxiety, 4 questions about curiosity and information needs, 3 questions about waiting time, 4 questions about prejudice, and 3 questions about mistrust. In the 5-point Likert-type PCCBS, the answers to the questions are categorized as “Strongly disagree”, “Disagree”, “Undecided”, “Agree”, and “Strongly agree”. In the scoring of the scale developed by Aydın and Şahin^(27)^, questions related to the need for attention, security personnel, fear and anxiety, curiosity and need for information, waiting time, and insecurity are scored on a scale of 1 to 5. In contrast, questions related to prejudice are scored in the opposite direction. Then, the total score for each subgroup was divided by the number of questions to calculate the subgroup score. Additionally, the total score of the entire scale was divided by the total number of questions to calculate the overall scale score. While the maximum score obtained from the scale is 140, it is thought that as the PCCBS average score increases, there are more obstacles to patient-centered communication. The Cronbach’s alpha value of the scale was calculated to be 0.81. For the sub-dimensions, Cronbach’s alpha values were 0.81 for need for attention, 0.78 for security personnel, 0.80 for fear and anxiety, 0.79 for curiosity and need for information, 0.75 for waiting time, 0.77 for prejudice, and 0.76 for distrust towards healthcare personnel^(20)^. In this study, the Cronbach alpha coefficient of the scale was 0.85. For the sub-dimensions, Cronbach’s alpha values were 0.72 for need for attention, 0.73 for security personnel, 0.84 for fear and anxiety, 0.90 for curiosity and need for information, 0.82 for waiting time, 0.71 for prejudice, and 0.82 for distrust towards healthcare personnel.

### Data Analysis

Statistical analyses were performed using the IBM Statistical Package for Social Sciences, Statistics for Windows, Version 24.0 (IBM Corp.; Armonk, NY, USA, Released 2016). Continuous variables were expressed as mean ± standard deviation (SD) (if normally distributed) or median [Interquartile Range (IQR) (Q1–Q3)] (if not normally distributed), and categorical variables were expressed as numbers and percentages. The normality of numerical variables was assessed using the Kolmogorov-Smirnov test. Descriptive analyses (number, percentage, mean, and standard deviation values) were used to determine the descriptive characteristics of the patients and the scores they obtained from the scales. Cronbach’s alpha reliability coefficient was calculated for each subscale and the total score to determine internal consistency. Mann-Whitney U and Kruskal-Wallis tests were used to examine differences between independent variables and total scores on the measurement tool. Linear regression analyses were conducted to identify factors associated with patient-centered communication barriers. Predictor variables were coded as follows: education was treated as an ordinal variable, income was categorized into three levels, and length of hospital stay was represented as a continuous variable; the reference category for categorical variables was the lowest category. Key regression assumptions were checked, including linearity, absence of multicollinearity (VIF < 10), regular distribution of residuals (Q-Q plot, Shapiro-Wilk test), homoscedasticity (Breusch-Pagan test), and independence of error terms (Durbin-Watson test). Outliers were assessed using Cook’s Distance. Residual plots and VIF values were examined to ensure model adequacy. If assumptions were violated, robust regression or ordinal models were considered. A p-value of less than 0.05 was considered statistically significant for all analyses. Multiple testing was adjusted using the False Discovery Rate method. The primary outcome of the study was the total score of the Patient-Centered Communication Barriers Scale (PCCBS).

### Ethical Aspects

The study was approved by the Ethics Review Board of the hospital (University of Health Sciences Ethical Committee Report, Approval no: 2022/95). After the volunteer participants were verbally informed about the study, all participants read and signed the informed consent form.

## RESULTS

A total of 336 participants were included in the study, with a mean age of 57.46 ± 17.32 years. Of the participants, 231 (68.7%) were male, 181 (53.9%) were married, 182 (54.2%) were in nuclear families, 231 (68.8%) had at least one child, and 268 (79.8%) had a relative taking care of them. 146 (43.5%) of the participants were primary school graduates, and 176 (52.4%) had income equal to expenses (Table 1).

**Table 1:** Sociodemographic characteristics of the participants (n = 336) – Ankara, Turkey, 2023.

Features	n	%
Age (years) Mean ± SD	57.46 ± 17.32	
**Gender**		
Men	231	68.7
Women	105	31.3
**Marital Status**		
Married	181	53.9
Single	155	46.1
**Education Status**		
Primary Education	146	43.5
High School	111	33.0
University and Above	79	23.5
**Income Level**		
Income Less Expenditure	123	36.6
Income Equal to Expenditure	176	52.4
Income More than Expenditure	37	11.0
**Family Structure**		
Nuclear Family	182	54.2
Extended Family	154	45.8
**Having a Child**		
Yes	231	68.8
No	105	31.2
**Caregiver**		
Present	268	79.8
No	68	20.2

*SD = Standard deviation.

Of the participants, 14.3% had lung cancer, and 13.1% had breast cancer. 73.2% had no additional disease. Ninety-one percent (91.1%) reported experiencing symptoms related to the disease, and 33.9% reported experiencing fatigue. The sociodemographic and clinical characteristics of the participants are summarized in Table 2.

**Table 2:** Clinical characteristics of the participants (n = 336) – Ankara, Turkey, 2023.

Features	n	%
Duration of hospitalization Mean ± SD	13.41 ± 15.92	
**Type of cancer**		
Lung	48	14.3
Breast	44	13.1
Testis	42	12.4
Ewing Sarcoma	38	11.2
Bowel	33	9.8
Prostate	30	8.9
Pancreatic	17	5.1
Central Nervous System	17	5.1
Liver	16	4.8
Stomach	16	4.8
Lymphoma	13	3.9
Other*	22	6.6
**Chronic diseases other than cancer**		
Yes	90	26.8
No	246	73.2
Comorbidities (n = 90)***		
Diabetes	17	18.9
Depression	13	14.4
Coronary Artery Disease	13	14.4
Hypertension	11	12.2
Vitamin Deficiency	11	12.2
Kidney failure	9	10.0
Other**	16	17.7
**Symptom Experience Status**		
Yes	306	91.1
No	30	8.9
Symptoms Experienced (n = 306)***		
Fatigue	114	33.9
Nausea	61	18.2
Fever	57	17.0
Pain	55	16.4
Diarrhea	38	11.3
Constipation	38	11.3
Mouth sores	35	10.4
Bleeding	34	10.1

*Kidney, esophagus, ovary, bladder, rectum, uterus, bile duct, nasopharynx, multiple myeloma.

**Cirrhosis, gastritis, chronic pain, glaucoma, hypothyroidism.

***n is folded, calculations are based on the n value.

Table 3 presents the mean scores of the Patient-Centered Communication Barriers Scale and its subdimensions. The total scale mean score was 3.69 ± 0.32. The mean scores for the subdimensions were as follows: Need for attention, 4.40 ± 0.44; Curiosity and need for information, 4.16 ± 0.81; Waiting time, 4.27 ± 0.95; Mistrust, 3.36 ± 0.61; Security personnel, 4.08 ± 0.78; Fear and anxiety, 3.31 ± 1.07; and Prejudice, 3.32 ± 0.81. Accordingly, among the participants, the highest levels of communication barriers were observed in the subdimensions of need for attention and waiting time. In contrast, the lowest levels were observed in the subdimensions of fear, anxiety, and prejudice.

**Table 3:** Distribution of mean scores of the patient-centered communication barriers scale and its subdimensions (n = 336) – Ankara, Turkey, 2023.

PCCBS	Mean ± SD	Min–Max	95% CI
Need for attention	4.40 ± 0.44	3.17–4.39	4.35–4.44
Curiosity and need for information	4.16 ± 0.81	2.25–5.0	4.07–4.24
Waiting time	4.27 ± 0.95	1.0–5.0	4.17–4.37
Mistrust	3.36 ± 0.61	3.0–5.0	4.33–4.45
Security personnel	4.08 ± 0.78	2.0–5.0	3.99–4.16
Fear and anxiety	3.31 ± 1.07	1.0–5.0	3.19–3.42
Prejudice	3.32 ± 0.81	2.0–5.0	3.23–3.30
Scale Total	3.69 ± 0.32	2.93–4.39	3.66–3.73

PCCBS: Patient-Centered Communication Barriers Scale.

In Table 4, scale scores were compared with sociodemographic and clinical characteristics. A statistical difference was found between the level of education, the presence of a caregiver, and experiencing symptoms (p < 0.05). It was found that the need for attention, information, and curiosity increased with increasing education levels, fear and anxiety increased in the absence of a caregiver, and the need for attention decreased in those who experienced symptoms.

**Table 4:** Comparison of patient-centered communication barriers scores with sociodemographic and clinical characteristics (n = 336) – Ankara, Turkey, 2023.

Variable	PCCBS scale total score	Need for attention	Curiosity and need for knowledge	Waiting time	Insecurity	Security personnel	Fear and anxiety	Prejudice

	Median (Q1–Q3)	Median (Q1–Q3)	Median (Q1–Q3)	Median (Q1–Q3)	Median (Q1–Q3)	Median (Q1–Q3)	Median (Q1–Q3)	Median (Q1–Q3)
**Gender**								
Men	3.7 (3.5–3.9)	4.3 (4.0–4.8)	4.2 (4.0–5.0)	4.6 (4.0–5.0)	4.3 (4.0–5.0)	4.2 (4.0–4.7)	3.2 (2.7–4.2)	3.2 (2.7–3.7)
Women	3.6 (3.5–3.9)	4.3 (4.0–4.8)	4.2 (3.5–5.0)	4.6 (4.0–5.0)	4.3 (4.0–5.0)	4.2 (4.0–4.5)	3.2 (3.0–4.0)	3.2 (2.7–3.7)
p*	0.854	0.267	0.719	0.671	0.354	0.279	0.965	0.922
**Marital Status**								
Married	3.6 (3.4–3.9)	4.4 (4.0–4.8)	4.2 (3.5–5.0)	4.3 (4.0–5.0)	4.3 (4.0–5.0)	4.2 (4.0–4.7)	3.2 (2.7–4.0)	3.5 (3.0–3.7)
Single	3.7 (3.5–3.9)	4.3 (4.0–4.8)	4.2 (4.0–5.0)	4.6 (4.0–5.0)	4.1 (4.0–5.0)	4.1 (4.0–4.5)	3.2 (2.8–4.2)	3.1 (2.7–3.7)
p*	0.708	0.740	0.451	0.279	0.797	0.331	0.653	0.288
**Education Status**								
Primary Education	3.6 (3.4–3.9)	4.3 (4.0–4.8)	4.0 (3.5–4.7)	4.6 (4.0–5.0)	4.3 (4.0–5.0)	4.3 (4.0–4.5)	3.2 (2.7–4.0)	3.2 (2.7–3.7)
High School	3.7 (3.5–3.9)	4.3 (4.0–4.8)	4.2 (4.0–5.0)	4.6 (4.0–5.0)	4.3 (4.0–5.0)	4.0 (3.7–4.6)	3.2 (3.0–4.2)	3.2 (2.8–3.7)
University and Above	3.7 (3.5–3.9)	4.6 (4.1–5.0)	4.7 (3.5–5.0)	4.6 (4.0–5.0)	4.3 (4.0–5.0)	4.2 (3.7–4.7)	3.2 (3.0–4.1)	3.2 (2.7–3.7)
p**	0.333	**0.004**a < b/η^2^ = 0.034	**0.005**a < b/η^2^ = 0.027	0.678	0.755	0.501	0.688	0.587
**Income Level**								
Income Less than Expenditure	3.6 (3.4–3.8)	4.1 (4.0–4.8)	4.0 (3.5–5.0)	4.3 (4.0–5.0)	4.3 (4.0–5.0)	4.0 (4.0–4.7)	3.2 (2.7–4.0)	3.2 (2.7–3.7)
Income Equal to Expenditure	3.7 (3.5–3.9)	4.3 (4.0–4.8)	4.2 (3.7–5.0)	4.6 (4.0–5.0)	4.3 (4.0–5.0)	4.2 (3.8–4.5)	3.2 (3.0–4.0)	3.2 (2.7–3.7)
Income More than Expenditure	3.8 (3.6–4.0)	4.5 (4.1–4.8)	4.2 (4.0–5.0)	4.6 (4.0–5.0)	4.3 (4.0–5.0)	4.2 (4.0–4.7)	3.5 (3.0–4.5)	3.7 (2.7–4.0)
p**	0.110	0.281	0.423	0.724	0.821	0.881	0.191	0.355
**Caregiver**								
Yes	3.7 (3.4–3.9)	4.3 (4.0–4.8)	4.2 (3.5–5.0)	4.6 (4.0–5.0)	4.3 (4.0–5.0)	4.2 (4.0–4.6)	3.2 (2.7–4.0)	3.2 (2.7–3.7)
No	3.7 (3.5–4.0)	4.1 (4.0–4.8)	4.2 (4.0–5.0)	4.6 (4.0–5.0)	4.3 (4.0–5.0)	4.2 (4.0–4.7)	4.0 (3.2–4.5)	3.5 (2.7–3.8)
p*	0.222	0.342	0.911	0.798	0.819	0.775	**0.001/η^2^ = 0.030**	0.655
**Symptom Experience Status**								
Yes	3.7 (3.5–3.9)	4.3 (4.0–4.8)	4.2 (3.5–5.0)	4.6 (4.0–5.0)	4.3 (4.0–5.0)	4.2 (4.0–4.7)	3.2 (2.7–4.0)	3.2 (2.7–3.7)
No	3.7 (3.6–3.9)	4.6 (4.1–5.0)	4.2 (4.0–5.0)	4.5 (4.0–5.0)	4.3 (4.0–5.0)	4.2 (4.0–4.7)	3.2 (2.7–4.0)	3.5 (3.0–4.0)
p*	0.361	**0.028**/η^2^ = 0.013	0.903	0.684	0.523	0.921	0.683	0.310

*Mann-Whitney U Test.

**Kruskal-Wallis Test.

^ab^Post hoc analysis.

η^2^ Eta Squared.

PCCBS: Patient-Centered Communication Barriers Scale.

Table 5 shows the effect of patient characteristics on patient-centered communication barriers. Regression analysis to determine the cause-and-effect relationship between the socio-economic and clinical characteristics of the participants and patient-centered communication barriers revealed significant results (p < 0.05; F = 7.843; F = 10.341; F = 5.712). The total change in the level of need for attention is explained by the level of education and symptom experience at a rate of 4.5% (R2 = 0.045). Participants with university or higher education had an increased need for attention and information (β = 0.175). Having symptoms decreases the need for attention (β = −0.116). The total change in the level of curiosity and need for information is explained by education level at a rate of 1.7% (R2 = 0.017). Having a university or higher education level was associated with greater curiosity and need for information (β = 0.130). The total change in fear and anxiety is explained by the absence of a caregiver at a rate of 3% (R2 = 0.030). The absence of a caregiver increases fear and anxiety (β = 0.173).

**Table 5:** The effect of patient characteristics on patient-centered communication barriers – Ankara, Turkey, 2023.

Independent variable	Unstandardized coefficients		Standardized coefficients		p	R2	95% Confidence interval	
	B	SE	β	t			Bottom	Top
Education Level* (University and Above)	0.098	0.030	0.175	3.258	0.001	0.045	0.039	0.157
Symptom Experience*	-0.181	0.084	-0.116	-2.162	0.031		-0.345	-0.016
Education Level** (University and Above)	0.133	0.056	0.130	2.390	0.017	0.017	0.024	0.242
Absence of a Caregiver***	0.465	0.144	0.173	5.216	0.001	0.030	0.180	0.749

*Dependent Variable = Need for Attention, R = 0.212; F = 7.834; Durbin-Watson = 1.888; df = 2.

**Dependent Variable = Curiosity and Need for Information, R = 0.130; F = 5.712; Durbin-Watson = 1.958; df = 1.

***Dependent Variable = Fear and Anxiety, R = 0.173; F = 10.341; Durbin-Watson = 1.967; df = 1.

β = coefficient of predictor variables; S.E. = Standard error.

## DISCUSSION

Effective communication is a cornerstone of patient-centered oncology care, as it plays a vital role in supporting patients’ understanding of their disease, promoting treatment adherence, enhancing psychological well-being, and ensuring satisfaction with care^(28)^. In this study, patients reported communication barriers most prominently in areas related to information needs and attention from healthcare professionals. Regression analyses demonstrated that education level and symptom experience were significant predictors of these barriers. Specifically, higher education was associated with greater curiosity, a higher need for information, and increased attention, while the presence of symptoms was linked to a decreased need for attention. In addition, the absence of a caregiver was associated with increased fear and anxiety.

Various factors influence communication between patients and healthcare professionals. These factors include the anxiety and concerns of patients and their relatives, needs for attention and information, mistrust and prejudice toward healthcare personnel, and long waiting times during the treatment process. Aydın’s study reported that relatives of patients had high levels of need for attention and curiosity/information-seeking, while their levels of prejudice were low. Additionally, relatives of hospitalized patients exhibited higher levels of curiosity/information-seeking and trust, whereas relatives of outpatients demonstrated higher levels of prejudice^(27)^. In the present study, the highest communication barriers were observed in the subdimensions of need for attention, curiosity and information- seeking, and waiting time among hospitalized patients. In contrast, the lowest barriers were found in the subdimensions of fear, anxiety, and prejudice. These findings are consistent with the literature, which emphasizes that patients’ information and attention needs are prioritized during the treatment process.

For example, Iacorossi et al.^(29)^ evaluated the quality of nurse–patient communication among hospitalized oncology patients and highlighted that meeting patients’ information and attention needs is critical for care satisfaction. Similarly, Primeau et al.^(30)^ systematic review showed that cancer patients demand empathetic and clear communication from multidisciplinary teams when receiving bad news. In addition to individual factors, cultural and systemic determinants also shape communication in oncology care. For example, recent evidence from Latin America underscores the importance of addressing communication barriers in this context. Studies from the region have shown that limited coordination within healthcare teams and insufficient patient–provider dialogue hinder effective communication in cancer care^(31)^. Similarly, culturally sensitive and community-based approaches have been found to strengthen trust and information exchange^(32)^. These findings align with our study, suggesting that communication barriers are influenced not only by individual factors such as education, symptoms, and caregiver presence but also by broader sociocultural and systemic dynamics. In this context, our study makes a significant contribution by systematically identifying the communication needs and barriers of patients and their relatives in the inpatient oncology setting at the subdimensional level, thereby addressing a gap in the literature. In particular, the prominence of the need for attention and waiting time highlights the necessity of interventions aimed at enhancing the active participation of patients and their relatives, as well as improving institutional processes in clinical practice. Furthermore, the literature emphasizes the importance of effective communication and interaction, as well as the provision of follow-up care protocols and guidelines. It is recommended that further research on patient-centered communication be integrated into clinical practice^(10,33)^. This study examines the communication barriers faced by patients and their relatives in the context of hospitalized oncology care in detail, thereby filling a gap in the literature and providing guidance for clinical practice.

This study found that those with university and higher education had more curiosity and information needs than those with high school and primary education. Information is the most critical need for cancer patients and can cause barriers to effective health communication^(34,35)^. Individuals with life-threatening diseases such as cancer want to know their prognosis, treatments, and side effects^(36)^. Similar to our study,research shows that the need for information about the disease increases with higher levels of education^(37,38)^. Individuals with higher levels of education tend to have higher levels of health literacy and better medical decision-making^(39)^, which enables them to communicate effectively with healthcare professionals^(40)^. Meeting patients’ information needs is critical for effective communication. Information can reduce anxiety and distress, support decision-making and patient satisfaction, and increase coping. Patients with advanced cancer often use information sources to understand the disease and maintain hope^(41,42)^. Recent population-based evidence from the United States also shows that higher levels of patient-centered communication are associated with fewer difficulties and less frustration during information-seeking among cancer survivors, emphasizing that both the availability of accurate information and the quality of communication shape patients’ experiences in navigating their disease^(43)^. Therefore, it is essential to inform patients about the issues they need to know in all processes, including diagnosis, treatment, and follow-up.

This study found that the need for attention increased with higher levels of education. Cancer patients require attention to ensure symptom control and to meet their psychosocial and economic needs^(44)^. The expectations of patients and their relatives from healthcare professionals, whether or not these expectations are met, have an impact on patients and their relatives. Limited attention to the needs of individuals with cancer can cause communication barriers^(45)^. Inadequate attention to patients’ needs may lead to unnecessary distress, increased healthcare costs, decreased quality of life, and patient dissatisfaction^(46)^. It is thought that the need for attention increases with education level because educated patients seek and follow correct sources of information about the disease process and its outcomes, enhancing their awareness of potential symptoms. For healthy communication, patients’ expectation of attention from healthcare personnel is considered a positive factor.

Surprisingly, this study found that the need for attention decreased as symptom status increased. Symptoms of advanced cancer patients are usually numerous, simultaneous, and severe. As the symptoms experienced by patients increase, their functional status declines, and their ability to perform daily activities and socialize decreases^(47)^. Supporting this, a recent study conducted in Türkiye found that patients with higher symptom burdens reported lower levels of patient-centered communication, highlighting the impact of physical and psychological distress on communication engagement^(28)^. Treiman et al.^(48)^ reported that colorectal cancer patients with poor physical health had lower mean scores in patient-centered communication and its sub-dimensions. Similarly, it was found that patients with poor health status had lower mean scores in patient-centered communication than those with good health status^(49)^. While cancer patients with poor health need enhanced patient-centered communication, disease-related barriers may impede interaction. Therefore, prioritizing patients with poorer health in interventions addressing survival needs may be essential for effective patient-centered communication^(42)^. Health professionals may assume that patients with poor physical status cannot engage in communication, which can significantly disrupt interaction^(49)^. The findings of this study suggest that patients experiencing intense complications such as fatigue, nausea, and pain from cancer treatment may be reluctant to communicate and prefer to rest. Therefore, it is essential to acknowledge severe symptoms and not ignore patients’ needs. Prioritizing patients with poor physical and mental health in patient-centered communication is recommended.

Coping with a life-threatening illness can trigger involuntary reactions such as anxiety, restlessness, hopelessness, anger, fear, depression, and helplessness^(47)^. The risk of experiencing anxiety is higher in the absence of a cohabiting person or with inadequate social support^(50)^. Considering the burden of the disease, especially in cancer patients, this is a critical issue to emphasize^(51)^. Family members or companions can also play a facilitating role in communication by helping patients obtain and understand information about the disease process^(10)^. In this study, it was found that patients’ fear and anxiety increased in the absence of a caregiver. Having a caregiver with cancer patients is essential for receiving information, accessing resources, managing treatment, and making informed decisions. It is thought that the absence of a support person during care, examinations, treatment, and hospitalization may cause patients to fear inadequacy in managing the cancer and treatment process. Nurses should therefore actively support patients in the absence of caregivers to reduce their fears and anxieties. Providing guidance and strategies to cope with the emotional and physical challenges of cancer is crucial to help patients reduce anxiety and enhance support for those who are alone. Recent research highlights that breakdowns in patient-centered communication can exacerbate these fears, especially when cancer survivors feel their informational and emotional needs are unmet^(52)^.

## CONCLUSION

Increased nurse-patient communication will have important implications for care outcomes and quality. Uncertainty can be a source of stress and anxiety for patients and can affect their ability to make informed decisions about their care. Nurses must help patients cope with uncertainty, identify situations that may create communication barriers during the disease process, meet the need for information and attention, and consider patients’ educational levels while providing all these. Considering that the need for attention of individuals with cancer-related symptoms may increase, the symptoms of patients should be evaluated during care. In severe symptoms that may be related to cancer and its treatment, the patient’s need for communication should not be ignored. The fears and concerns of patients who are alone, i.e., those with no one to care for them, should be identified, and their needs should be addressed.

This study should be interpreted in light of certain limitations. Its cross-sectional design precludes causal inferences, and the single-center sample may limit the generalizability of findings. Furthermore, although numerical inconsistencies have been corrected, reporting bias cannot be entirely excluded. Finally, the use of self-reported data may introduce recall or social desirability bias. Despite these limitations, this study provides valuable insights into patient-centered communication barriers among oncology patients and highlights key areas for clinical improvement. These findings also underscore the importance of targeted communication training and literacy- sensitive interventions for nurses, suggesting that future longitudinal studies could further explore the causal relationships between patient characteristics and communication barriers in oncology care.

## Data Availability

The entire dataset supporting the results of this study is available upon request to the corresponding author.
