# Supplementary material for: Barriers and factors associated with patient-centered communication in oncology inpatients: a cross-sectional study
Source: Rev Esc Enferm USP. 2026 Nov 6;60:e20250265. doi: 10.1590/1980-220X-REEUSP-2025-0265en (PMC13743176; doi:10.1590/1980-220X-REEUSP-2025-0265en)
Supplement: Translation

# Translation: Barreras y factores asociados con la comunicación centrada en el paciente en pacientes hospitalizados de oncología: un estudio transversal

**DOI:** 10.1590/1980-220X-REEUSP-2025-0265es

**Authors:** Betülay Kiliç, Serap Korkmaz, Selcan Erel Kurt, Fatma İlknur Çinar

**Affiliations:** 1 University of Health Sciences, Gulhane Faculty of Nursing, Ankara, Türkiye.; 2 Cyprus International University, Faculty of Health Sciences, Lefkoşa, Türkiye.

**Keywords:** Barreras de Comunicación, Relaciones Enfermera-Paciente, Enfermería Oncológica, Atención Dirigida al Paciente

## Abstract

**Objetivo:**

El objetivo de este estudio fue evaluar las barreras a la comunicación centrada en el paciente en pacientes oncológicos y los factores que influyen en dichas barreras.

**Métodos:**

Se llevó a cabo un estudio transversal con 336 pacientes con cáncer hospitalizados (≥18 años) en la Clínica de Oncología de un hospital de formación e investigación entre febrero de 2022 y marzo de 2023. Se excluyó a los pacientes con demencia o discapacidad intelectual. Los datos se recopilaron mediante entrevistas presenciales utilizando un formulario sociodemográfico y la Escala de Barreras de Comunicación Centrada en el Paciente (Patient-Centered Communication Barriers Scale – PCCBS).

**Resultados:**

La edad promedio de los participantes fue de 57,5 ± 17,3 años; el 68,7% eran hombres, y el período promedio de hospitalización fue de 13,4 ± 15,9 días. El análisis de regresión mostró relaciones significativas entre las características de los pacientes y las barreras de comunicación. Un mayor nivel educativo aumentó la necesidad de atención e información de los pacientes (β = 0,175, p = 0,001, IC del 95% [0,039–0,157]) y su curiosidad (β = 0,130, p = 0,017, IC del 95% [[0,024–0,242]). Una mayor carga de síntomas reducía la necesidad de atención (β = –0,116, p = 0,031, IC del 95% [–0,345, –0,016]). La ausencia de un cuidador se asoció con mayores niveles de miedo y ansiedad (β = 0,173, p = 0,001, IC del 95% [0,180–0,749]). El nivel educativo y la experiencia con los síntomas explicaron el 4,5% de la varianza en la necesidad de atención (R2 = 0,045), el 1,7% en la curiosidad y las necesidades de información (R2 = 0,017) y el 3% en el miedo y la ansiedad (R2 = 0,030).

**Conclusión:**

Los pacientes con mayor nivel educativo reportaron mayores necesidades de información, mientras que aquellos con una mayor carga de síntomas eran menos propensos a buscar atención. La falta de apoyo por parte de los cuidadores aumentaba el miedo y la ansiedad. Estos resultados indican que las barreras de comunicación en oncología se ven influidas por el nivel educativo, la experiencia con los síntomas y el contexto de cuidados. Las enfermeras y los médicos deben evaluar estos factores de manera sistemática y adaptar las estrategias de comunicación a las necesidades individuales. La implementación de tales intervenciones y la detección de barreras pueden mejorar la atención oncológica centrada en el paciente. Sin embargo, el diseño transversal y unicéntrico limita la inferencia causal y la generalizabilidad.

## INTRODUCCIÓN

El cáncer es un problema de salud devastador que da lugar a diferentes experiencias según la respuesta de los pacientes al tratamiento a lo largo del proceso de diagnóstico y tratamiento. Sigue siendo una de las principales causas de morbilidad y mortalidad en todo el mundo, con aproximadamente 20 millones de casos nuevos y 9,7 millones de muertes reportadas anualmente. Estas cifras ilustran la carga global del cáncer y subrayan la importancia de una comunicación eficaz en la atención oncológica para abordar las necesidades psicológicas e informativas de los pacientes^(1)^. La comunicación en la atención oncológica es crucial debido a la naturaleza delicada de la información y a su profundo impacto psicosocial en los pacientes y sus familias^(2)^. Una comunicación eficaz puede motivar la prevención de enfermedades, el cambio de comportamiento y la promoción de la salud^(3)^. Más allá del proceso de tratamiento, una de las funciones esenciales del equipo de salud es garantizar que los mensajes transmitidos sean coherentes con las necesidades y preferencias individuales del paciente^(4)^.

La comunicación centrada en el paciente puede contribuir directa e indirectamente a identificar las necesidades y preocupaciones de los pacientes, así como a su comprensión, satisfacción, motivación, confianza y salud general^(5,6)^. Este enfoque es crucial para brindar atención oncológica de alta calidad y puede ser eficaz de diversas maneras, incluyendo la promoción de la salud, la protección y la prevención de enfermedades. La comunicación eficaz en materia de salud puede ayudar a los pacientes y a sus familias a tomar decisiones informadas, sobrellevar el cáncer y manejar el proceso de atención de manera efectiva^(7)^. Por otro lado, una comunicación inadecuada puede provocar interrupciones en la atención, el rechazo al tratamiento y diversos errores^(6)^. Además, mejora la calidad de la atención médica y contribuye a tomar precauciones al aumentar la conciencia de las personas sobre el riesgo de enfermedad^(5,8)^.

Comprender y empatizar con las perspectivas de los pacientes sobre la enfermedad son componentes fundamentales de la comunicación centrada en el paciente^(9)^. Este enfoque de comunicación revela las inquietudes, ideas, expectativas, necesidades, emociones y el entorno psicosocial y cultural único de los pacientes. Además, el paciente y el equipo de salud pueden establecer un entendimiento compartido de los tratamientos que se alineen con los valores y preferencias del paciente^(10)^. La comunicación efectiva entre los pacientes y los proveedores aumenta la participación de los pacientes en los servicios de salud y tiene un impacto positivo en la calidad del servicio y los resultados de salud^(11)^.

La comunicación centrada en el paciente requiere que las enfermeras desarrollen una comprensión compartida del problema, los objetivos del tratamiento y las barreras para la salud. Este enfoque implica aceptar la personalidad, la historia de vida y el contexto social^(12)^ de la persona. La comunicación centrada en el paciente se refiere a los procesos y resultados de las interacciones entre enfermeras, proveedores de servicios y pacientes. La calidad de los servicios de salud depende de la relación entre el paciente y el profesional de la salud. Las enfermeras, en particular, desempeñan un papel fundamental en la atención centrada en el paciente, y el éxito de esta atención también depende de su comunicación efectiva con los pacientes^(13)^. Se ha demostrado que la comunicación centrada en el paciente evita que los pacientes abandonen el tratamiento y genera mejores resultados clínicos^(14,15)^. En la literatura existente, factores como la educación del paciente, la falta de información, la carga de los síntomas y el apoyo de los cuidadores se asocian con barreras de comunicación^(16–18)^. Además, algunos estudios han revelado que los factores relacionados con el paciente y la familia se consideran las barreras más significativas para las conversaciones sobre los objetivos de la atención^(19)^. Al mismo tiempo, marcos recientes, como la Escala de Barreras de Comunicación Centrada en el Paciente (Patient-Centered Communication Barriers Scale – PCCBS), destacan que las barreras de comunicación se presentan en múltiples ámbitos de la interacción entre el paciente y el proveedor de atención^(20)^. Cada ámbito refleja un desafío distinto para la implementación de los principios de la atención centrada en el paciente.

Por ejemplo, la percepción de falta de atención puede reforzar la sensación de los pacientes de ser subestimados, lo que socava la confianza y la comunicación abierta^(21)^. El miedo y la ansiedad pueden impedir que los pacientes hagan preguntas o participen activamente en el proceso de tratamiento, lo que los coloca en un papel pasivo. Del mismo modo, la curiosidad insatisfecha y las necesidades de información no cubiertas dificultan que los pacientes comprendan su afección y su tratamiento, lo que genera incertidumbre, malinterpretaciones y brechas de comunicación^(22)^. Los factores ambientales y organizacionales también desempeñan un papel significativo; los largos tiempos de espera pueden provocar impaciencia, fatiga y frustración, mientras que las interacciones negativas con el personal de seguridad pueden disminuir la sensación de comodidad y seguridad de los pacientes, lo que en última instancia afecta la comunicación^(23)^. Por último, los prejuicios y la desconfianza pueden obstaculizar el establecimiento de una relación terapéutica, lo que debilita la colaboración en el tratamiento^(24)^. Entre las barreras a las que se enfrentan las enfermeras oncológicas en la comunicación centrada en el paciente, destacan especialmente la falta de tiempo, las deficiencias en la comunicación del equipo y las habilidades limitadas de empatía^(5)^. Sin embargo, aún no se han investigado suficientemente el alcance y el contexto de estas barreras entre los pacientes oncológicos hospitalizados.

Este estudio tiene como objetivo evaluar sistemáticamente las barreras a la comunicación centrada en el paciente y los factores relacionados en pacientes oncológicos hospitalizados, contribuyendo así a la mejora de la práctica clínica.

## MÉTODOS

### Diseño del Estudio y Muestra

Este estudio transversal se llevó a cabo con 336 pacientes con cáncer hospitalizados en la Clínica de Oncología de un hospital de formación e investigación entre febrero de 2022 y marzo de 2023. El número total de pacientes que acudieron a la Clínica de Oncología fue de 2990, incluyendo 524 reingresos. Para evitar contar a los mismos pacientes varias veces, se consideró que el tamaño adecuado de la población era de 2466. El tamaño de la muestra se calculó utilizando la fórmula establecida por Salant y Dillman (1994):


$$n=\frac{Nt^{2}pq}{d^{2}\left(N-1\right)+t^{2}pq}$$

N: Tamaño total de la población.

n: Número de individuos a muestrear.

p: Frecuencia de ocurrencia del evento estudiado (probabilidad de ocurrencia).

q: Frecuencia de no ocurrencia del evento estudiado (probabilidad de no ocurrencia).

t: Valor teórico de la tabla t correspondiente a un nivel de significación dado.

d: Margen de error aceptado para la frecuencia de ocurrencia del evento.

El tamaño de la muestra se calculó utilizando la fórmula de Salant y Dillman para esta población no homogénea con un intervalo de confianza del 95% y un error de muestreo de ± 5%, y el tamaño de muestra requerido fue n = 2466 (1,96)2 (0,50) (0,50)/(0,05)2 (2466 − 1) + (1,96)2 (0,50) (0,50) = 332 participantes^(25)^. Los participantes fueron reclutados mediante un método de muestreo consecutivo. Durante el período de recolección de datos, se invitó de manera consecutiva al estudio a todos los pacientes hospitalizados que cumplían con los criterios de inclusión y aceptaron participar. Se incluyeron a personas mayores de 18 años, diagnosticadas con cáncer y que aceptaron participar. Se excluyó en la selección a los pacientes con un diagnóstico conocido de demencia o discapacidad intelectual. Se recopilaron datos de 340 pacientes; sin embargo, se excluyó a 4 pacientes del análisis debido a datos faltantes, lo que dio como resultado una muestra final de 336 participantes. Se utilizaron las directrices “Strengthening the Reporting of Observational Studies in Epidemiology” (STROBE) como guía para garantizar el rigor metodológico^(26)^.

#### Participantes

- Pacientes hospitalizados en la unidad de oncología y pacientes ambulatorios que reciben quimioterapia (n = 340).
- Excluidos: datos faltantes o incompletos (n = 4).
- Muestra final incluida en el análisis (n = 336).

### Recopilación de Datos

Los datos se recopilaron mediante el “Formulario sociodemográfico” y la “Escala de barreras de comunicación centrada en el paciente (PCCBS)”. Los investigadores recopilaron los datos mediante entrevistas presenciales. Se pidió a los pacientes que firmaran un formulario de consentimiento informado antes de la recopilación de datos. La recopilación de datos duró aproximadamente entre 7 y 8 minutos por cada paciente.

### El Formulario Sociodemográfico

El formulario sociodemográfico que completaron los participantes del estudio constaba de 18 preguntas. Incluía preguntas sobre el año de nacimiento, el género, el estado civil, el nivel de ingresos, el número de hijos, el nivel educativo, el tipo de cáncer, la presencia de comorbilidades, la duración de la hospitalización y los síntomas experimentados.

### Escala de Barreras de Comunicación Centrada en el Paciente (PCCBS)

La escala de barreras de comunicación centrada en el paciente comprende 28 preguntas organizadas en siete subgrupos: necesidad de atención, personal de seguridad, miedo y ansiedad, curiosidad y necesidad de información, tiempo de espera, prejuicios y desconfianza. La escala consta de 6 preguntas sobre la necesidad de atención, 4 preguntas sobre el personal de seguridad, 4 preguntas sobre el miedo y la ansiedad, 4 preguntas sobre la curiosidad y la necesidad de información, 3 preguntas sobre el tiempo de espera, 4 preguntas sobre los prejuicios y 3 preguntas sobre la desconfianza. En la escala PCCBS de tipo Likert de 5 puntos, las respuestas a las preguntas se clasifican como “Totalmente en desacuerdo”, “En desacuerdo”, “Indeciso”, “De acuerdo” y “Totalmente de acuerdo”. En la puntuación de la escala desarrollada por Aydın y Şahin^(27)^, las preguntas relacionadas con la necesidad de atención, el personal de seguridad, el miedo y la ansiedad, la curiosidad y la necesidad de información, el tiempo de espera y la inseguridad se califican en una escala del 1 al 5. Por el contrario, las preguntas relacionadas con los prejuicios se califican en sentido contrario. Luego, la puntuación total de cada subgrupo se dividió por el número de preguntas para calcular la puntuación del subgrupo. Además, la puntuación total de toda la escala se dividió entre el número total de preguntas para calcular la puntuación general de la escala. Si bien la puntuación máxima que se puede obtener en la escala es de 140, se considera que, a medida que aumenta la puntuación promedio de la PCCBS, surgen más obstáculos para la comunicación centrada en el paciente. Se calculó que el valor alfa de Cronbach de la escala fue de 0,81. En cuanto a las subdimensiones, los valores del alfa de Cronbach fueron de 0,81 para la necesidad de atención, 0,78 para el personal de seguridad, 0,80 para el miedo y la ansiedad, 0,79 para la curiosidad y la necesidad de información, 0,75 para el tiempo de espera, 0,77 para los prejuicios y 0,76 para la desconfianza hacia el personal de salud^(20)^. En este estudio, el coeficiente alfa de Cronbach de la escala fue de 0,85. En cuanto a las subdimensiones, los valores del alfa de Cronbach fueron de 0,72 para la necesidad de atención, 0,73 para el personal de seguridad, 0,84 para el miedo y la ansiedad, 0,90 para la curiosidad y la necesidad de información, 0,82 para el tiempo de espera, 0,71 para los prejuicios y 0,82 para la desconfianza hacia el personal de salud.

### Análisis de Datos

Los análisis estadísticos se realizaron utilizando el Paquete Estadístico de IBM para Ciencias Sociales, Statistics for Windows, versión 24.0 (IBM Corp.; Armonk, NY, EE. UU., lanzado en 2016). Las variables continuas se expresaron como media ± desviación estándar (DE) (si seguían una distribución normal) o mediana [rango intercuartílico (IQR) (Q1–Q3)] (si no seguían una distribución normal), y las variables categóricas se expresaron como números y porcentajes. La normalidad de las variables numéricas se evaluó mediante la prueba de Kolmogorov-Smirnov. Se utilizaron análisis descriptivos (número, porcentaje, media y desviación estándar) para determinar las características descriptivas de los pacientes y las puntuaciones que obtuvieron en las escalas. Se calculó el coeficiente de confiabilidad alfa de Cronbach para cada subescala y para la puntuación total a fin de determinar la consistencia interna. Se utilizaron las pruebas U de Mann-Whitney y de Kruskal-Wallis para examinar las diferencias entre las variables independientes y las puntuaciones totales en la herramienta de medición. Se realizaron análisis de regresión lineal para identificar los factores asociados con las barreras de comunicación centradas en el paciente. Las variables predictoras se codificaron de la siguiente manera: el nivel educativo se trató como una variable ordinal, los ingresos se clasificaron en tres niveles y la duración de la estancia hospitalaria se representó como una variable continua; la categoría de referencia para las variables categóricas fue la categoría más baja. Se verificaron los supuestos clave de la regresión, incluyendo la linealidad, la ausencia de multicolinealidad (VIF < 10), la distribución normal de los residuos (gráfico Q-Q, prueba de Shapiro-Wilk), la homocedasticidad (prueba de Breusch-Pagan) y la independencia de los términos de error (prueba de Durbin-Watson). Los valores atípicos se evaluaron utilizando la distancia de Cook. Se examinaron los gráficos de residuos y los valores de VIF para garantizar la adecuación del modelo. En caso de incumplimiento de los supuestos, se consideraron modelos de regresión robustos o modelos ordinales. Se consideró estadísticamente significativo un valor p menor a 0,05 para todos los análisis. Las pruebas múltiples se ajustaron utilizando el método de la tasa de descubrimientos falsos. El resultado principal del estudio fue el puntaje total de la Escala de Barreras de Comunicación Centrada en el Paciente (Patient-Centered Communication Barriers Scale – PCCBS).

### Aspectos Éticos

El estudio fue aprobado por la Junta de Revisión Ética del hospital (Informe del Comité de Ética de la Universidad de Ciencias de la Salud, N.º de aprobación: 2022/95). Después de que se informara verbalmente a los participantes voluntarios sobre el estudio, todos leyeron y firmaron el formulario de consentimiento informado.

## RESULTADOS

Se incluyó en el estudio a un total de 336 participantes, con una edad promedio de 57,46 ± 17,32 años. De los participantes, 231 (68,7%) eran hombres, 181 (53,9%) estaban casados, 182 (54,2%) vivían en familias nucleares, 231 (68,8%) tenían al menos un hijo y 268 (79,8%) contaban con un familiar que los cuidaba. 146 (43,5%) de los participantes habían terminado la escuela primaria, y 176 (52,4%) tenían ingresos iguales a sus gastos (Tabla 1).

**Tabla 1:** Características sociodemográficas de los participantes (n = 336) – Ankara, Turquía, 2023.

Características	n	%
Edad (años) Media ± DE	57,46 ± 17,32	
**Sexo**		
Hombres	231	68,7
Mujeres	105	31,3
**Estado civil**		
Casado	181	53,9
Soltero	155	46,1
**Nivel educativo**		
Educación primaria	146	43,5
Educación secundaria	111	33,0
Universitaria y superior	79	23,5
**Nivel de ingresos**		
Ingresos menos gastos	123	36,6
Ingresos iguales a los gastos	176	52,4
Ingresos superiores a los gastos	37	11,0
**Estructura familiar**		
Familia nuclear	182	54,2
Familia extensa	154	45,8
**Tener un hijo**		
Sí	231	68,8
No	105	31,2
**Cuidador**		
Presente	268	79,8
No	68	20,2

*DE = Desviación estándar.

De los participantes, el 14,3% padecía cáncer de pulmón y el 13,1%, cáncer de mama. El 73,2% no padecía ninguna otra enfermedad. El 91,1% declaró experimentar síntomas relacionados con la enfermedad, y el 33,9% declaró sufrir fatiga. Las características sociodemográficas y clínicas de los participantes se resumen en la Tabla 2.

**Tabla 2:** Características clínicas de los participantes (n = 336) – Ankara, Turquía, 2023.

Características	n	%
Duración de la hospitalización Media ± DE	13,41 ± 15,92	
**Tipo de cáncer**		
Pulmón	48	14,3
De mama	44	13,1
De testículo	42	12,4
Sarcoma de Ewing	38	11,2
Intestino	33	9,8
Próstata	30	8,9
Páncreas	17	5,1
Sistema nervioso central	17	5,1
Hígado	16	4,8
Estómago	16	4,8
Linfoma	13	3,9
Otros*	22	6,6
**Enfermedades crónicas distintas del cáncer**		
Sí	90	26,8
No	246	73,2
Comorbilidades (n = 90)***		
Diabetes	17	18,9
Depresión	13	14,4
Enfermedad coronaria	13	14,4
Hipertensión	11	12,2
Deficiencia de vitaminas	11	12,2
Insuficiencia renal	9	10,0
Otros**	16	17,7
**Estado de los síntomas**		
Sí	306	91,1
No	30	8,9
Síntomas experimentados (n = 306)***		
Fatiga	114	33,9
Náuseas	61	18,2
Fiebre	57	17,0
Dolor	55	16,4
Diarrea	38	11,3
Estreñimiento	38	11,3
Llagas en la boca	35	10,4
Sangrado	34	10,1

*Riñón, esófago, ovario, vejiga, recto, útero, conducto biliar, nasofaringe, mieloma múltiple.

**Cirrosis, gastritis, dolor crónico, glaucoma, hipotiroidismo.

***n está agrupado; los cálculos se basan en el valor de n.

La Tabla 3 presenta las puntuaciones medias de la Escala de Barreras en la Comunicación Centrada en el Paciente y sus subdimensiones. La puntuación media total de la escala fue de 3,69 ± 0,32. Las puntuaciones medias de las subdimensiones fueron las siguientes: Necesidad de atención, 4,40 ± 0,44; Curiosidad y necesidad de información, 4,16 ± 0,81; Tiempo de espera, 4,27 ± 0,95; Desconfianza, 3,36 ± 0,61; Personal de seguridad, 4,08 ± 0,78; Miedo y ansiedad, 3,31 ± 1,07; y Prejuicio, 3,32 ± 0,81. En consecuencia, entre los participantes se observaron los niveles más altos de barreras comunicativas en las subdimensiones de necesidad de atención y tiempo de espera. Por el contrario, los niveles más bajos se observaron en las subdimensiones de miedo, ansiedad y prejuicio.

**Tabla 3:** Distribución de las puntuaciones medias de la escala de barreras de comunicación centrada en el paciente y sus subdimensiones (n = 336) – Ankara, Turquía, 2023.

PCCBS	Media ± DE	Mín.–Máx.	IC del 95%
Necesidad de atención	4,40 ± 0,44	3,17–4,39	4,35–4,44
Curiosidad y necesidad de información	4,16 ± 0,81	2,25–5,0	4,07–4,24
Tiempo de espera	4,27 ± 0,95	1,0–5,0	4,17–4,37
Desconfianza	3,36 ± 0,61	3,0–5,0	4,33–4,45
Personal de seguridad	4,08 ± 0,78	2,0–5,0	3,99–4,16
Miedo y ansiedad	3,31 ± 1,07	1,0–5,0	3,19–3,42
Prejuicio	3,32 ± 0,81	2,0–5,0	3,23–3,30
Total de la escala	3,69 ± 0,32	2,93–4,39	3,66–3,73

PCCBS: Escala de Barreras en la Comunicación Centrada en el Paciente.

En la Tabla 4 se compararon las puntuaciones de la escala con las características sociodemográficas y clínicas. Se observó una diferencia estadística entre el nivel de estudios, la presencia de un cuidador y la presencia de síntomas (p < 0,05). Se observó que la necesidad de atención, de información y la curiosidad aumentaban a medida que aumentaba el nivel de estudios; el miedo y la ansiedad aumentaban en ausencia de un cuidador; y la necesidad de atención disminuía en quienes.

**Tabla 4:** Comparación de las puntuaciones de la escala de barreras en la comunicación centrada en el paciente con las características sociodemográficas y clínicas (n = 336) – Ankara, Turquía, 2023.

Variable	Puntuación total de la escala PCCBS	Necesidad de atención	Curiosidad y necesidad de conocimiento	Tiempo de espera	Inseguridad	Personal de seguridad	Miedo y ansiedad	Prejuicio

	Mediana (Q1–Q3)	Mediana (Q1–Q3)	Mediana (Q1–Q3)	Mediana (Q1–Q3)	Mediana (Q1–Q3)	Mediana (Q1–Q3)	Mediana (Q1–Q3)	Mediana (Q1–Q3)
**Género**								
Hombres	3,7 (3,5–3,9)	4,3 (4,0–4,8)	4,2 (4,0–5,0)	4,6 (4,0–5,0)	4,3 (4,0–5,0)	4,2 (4,0–4,7)	3,2 (2,7–4,2)	3,2 (2,7–3,7)
Mujeres	3,6 (3,5–3,9)	4,3 (4,0–4,8)	4,2 (3,5–5,0)	4,6 (4,0–5,0)	4,3 (4,0–5,0)	4,2 (4,0–4,5)	3,2 (3,0–4,0)	3,2 (2,7–3,7)
p*	0,854	0,267	0,719	0,671	0,354	0,279	0,965	0,922
**Estado civil**								
Casado	3,6 (3,4–3,9)	4,4 (4,0–4,8)	4,2 (3,5–5,0)	4,3 (4,0–5,0)	4,3 (4,0–5,0)	4,2 (4,0–4,7)	3,2 (2,7–4,0)	3,5 (3,0–3,7)
Soltero	3,7 (3,5–3,9)	4,3 (4,0–4,8)	4,2 (4,0–5,0)	4,6 (4,0–5,0)	4,1 (4,0–5,0)	4,1 (4,0–4,5)	3,2 (2,8–4,2)	3,1 (2,7–3,7)
p*	0,708	0,740	0,451	0,279	0,797	0,331	0,653	0,288
**Nivel educativo**								
Educación primaria	3,6 (3,4–3,9)	4,3 (4,0–4,8)	4,0 (3,5–4,7)	4,6 (4,0–5,0)	4,3 (4,0–5,0)	4,3 (4,0–4,5)	3,2 (2,7–4,0)	3,2 (2,7–3,7)
Educación secundaria	3,7 (3,5–3,9)	4,3 (4,0–4,8)	4,2 (4,0–5,0)	4,6 (4,0–5,0)	4,3 (4,0–5,0)	4,0 (3,7–4,6)	3,2 (3,0–4,2)	3,2 (2,8–3,7)
Universidad y niveles superiores	3,7 (3,5–3,9)	4,6 (4,1–5,0)	4,7 (3,5–5,0)	4,6 (4,0–5,0)	4,3 (4,0–5,0)	4,2 (3,7–4,7)	3,2 (3,0–4,1)	3,2 (2,7–3,7)
p**	0,333	**0,004** **a < b / η^2^ = 0,034**	**0,005** **a < b / η^2^ = 0,027**	0,678	0,755	0,501	0,688	0,587
**Nivel de ingresos**								
Ingresos inferiores a los gastos	3,6 (3,4–3,8)	4,1 (4,0–4,8)	4,0 (3,5–5,0)	4,3 (4,0–5,0)	4,3 (4,0–5,0)	4,0 (4,0–4,7)	3,2 (2,7–4,0)	3,2 (2,7–3,7)
Ingresos iguales a los gastos	3,7 (3,5–3,9)	4,3 (4,0–4,8)	4,2 (3,7–5,0)	4,6 (4,0–5,0)	4,3 (4,0–5,0)	4,2 (3,8–4,5)	3,2 (3,0–4,0)	3,2 (2,7–3,7)
Ingresos superiores a los gastos	3,8 (3,6–4,0)	4,5 (4,1–4,8)	4,2 (4,0–5,0)	4,6 (4,0–5,0)	4,3 (4,0–5,0)	4,2 (4,0–4,7)	3,5 (3,0–4,5)	3,7 (2,7–4,0)
p**	0,110	0,281	0,423	0,724	0,821	0,881	0,191	0,355
**Cuidador**								
Sí	3,7 (3,4–3,9)	4,3 (4,0–4,8)	4,2 (3,5–5,0)	4,6 (4,0–5,0)	4,3 (4,0–5,0)	4,2 (4,0–4,6)	3,2 (2,7–4,0)	3,2 (2,7–3,7)
No	3,7 (3,5–4,0)	4,1 (4,0–4,8)	4,2 (4,0–5,0)	4,6 (4,0–5,0)	4,3 (4,0–5,0)	4,2 (4,0–4,7)	4,0 (3,2–4,5)	3,5 (2,7–3,8)
p*	0,222	0,342	0,911	0,798	0,819	0,775	**0,001 / η^2^ = 0,030**	0,655
**Estado de la experiencia de los síntomas**								
Sí	3,7 (3,5–3,9)	4,3 (4,0–4,8)	4,2 (3,5–5,0)	4,6 (4,0–5,0)	4,3 (4,0–5,0)	4,2 (4,0–4,7)	3,2 (2,7–4,0)	3,2 (2,7–3,7)
No	3,7 (3,6–3,9)	4,6 (4,1–5,0)	4,2 (4,0–5,0)	4,5 (4,0–5,0)	4,3 (4,0–5,0)	4,2 (4,0–4,7)	3,2 (2,7–4,0)	3,5 (3,0–4,0)
p*	0,361	**0,028/η^2^ = 0,013**	0,903	0,684	0,523	0,921	0,683	0,310

*Prueba U de Mann-Whitney.

**Prueba de Kruskal-Wallis.

^ab^Análisis post hoc.

η^2^ Eta al cuadrado.

PCCBS: Escala de Barreras en la Comunicación Centrada en el Paciente.

La tabla 5 muestra el efecto de las características de los pacientes sobre las barreras de comunicación centrada en el paciente. El análisis de regresión para determinar la relación de causa y efecto entre las características socioeconómicas y clínicas de los participantes y las barreras de comunicación centrada en el paciente reveló resultados significativos (p < 0,05; F = 7,843; F = 10,341; F = 5,712). El cambio total en el nivel de necesidad de atención se explica por el nivel de estudios y la experiencia de los síntomas en un 4,5% (R2 = 0,045). Los participantes con estudios universitarios o superiores presentaban una mayor necesidad de atención e información (β = 0,175). La presencia de síntomas reduce la necesidad de atención (β = −0,116). El cambio total en el nivel de curiosidad y necesidad de información se explica por el nivel educativo en un 1,7% (R2 = 0,017). Tener un nivel de estudios universitarios o superiores se asoció con una mayor curiosidad y necesidad de información (β = 0,130). El cambio total en el miedo y la ansiedad se explica por la ausencia de un cuidador en un 3% (R2 = 0,030). La ausencia de un cuidador aumenta el miedo y la ansiedad (β = 0,173).

**Tabla 5:** Efecto de las características de los pacientes sobre las barreras de la comunicación centrada en el paciente – Ankara, Turquía, 2023.

Variable independiente	Coeficientes no estandarizados		Coeficientes estandarizados		p	R^2^	Intervalo de confianza del 95%	
	B	SE	β	t			Parte inferior	Arriba
Nivel educativo* (universitario y superior)	0,098	0,030	0,175	3,258	0,001	0,045	0,039	0,157
Experiencia de los síntomas*	–0,181	0,084	–0,116	–2,162	0,031	–0,345	–0,016
Nivel educativo** (Universitario y superior)	0,133	0,056	0,130	2,390	0,017	0,017	0,024	0,242
Ausencia de un cuidador***	0,465	0,144	0,173	5,216	0,001	0,030	0,180	0,749

*Variable dependiente = Necesidad de atención, R = 0,212; F = 7,834; Durbin-Watson = 1,888; df = 2.

**Variable dependiente = Curiosidad y necesidad de información, R = 0,130; F = 5,712; Durbin-Watson = 1,958; df = 1.

***Variable dependiente = Miedo y ansiedad, R = 0,173; F = 10,341; Durbin-Watson = 1,967; df = 1.

β = coeficiente de las variables predictoras; E.E. = error estândar.

## DISCUSIÓN

La comunicación efectiva es un pilar fundamental de la atención oncológica centrada en el paciente, ya que desempeña un papel vital para ayudar a los pacientes a comprender su enfermedad, promover el cumplimiento del tratamiento, mejorar el bienestar psicológico y garantizar la satisfacción con la atención^(28)^. En este estudio, los pacientes señalaron que las barreras de comunicación se manifestaban de manera más prominente en áreas relacionadas con las necesidades de información y la atención por parte de los profesionales de la salud. Los análisis de regresión demostraron que el nivel educativo y la experiencia de los síntomas eran predictores significativos de estas barreras. Específicamente, un mayor nivel educativo se asoció con mayor curiosidad, una mayor necesidad de información y mayor atención, mientras que la presencia de síntomas se relacionó con una menor necesidad de atención. Además, la ausencia de un cuidador se asoció con un aumento del miedo y la ansiedad.

Varios factores influyen en la comunicación entre los pacientes y los profesionales de la salud. Estos factores incluyen la ansiedad y las preocupaciones de los pacientes y sus familiares, las necesidades de atención e información, la desconfianza y los prejuicios hacia el personal de salud, y los largos tiempos de espera durante el proceso de tratamiento. El estudio de Aydın informó que los familiares de los pacientes presentaban altos niveles de necesidad de atención y curiosidad/búsqueda de información, mientras que sus niveles de prejuicio eran bajos. Además, los familiares de pacientes hospitalizados mostraron mayores niveles de curiosidad/búsqueda de información y confianza, en tanto que los familiares de pacientes ambulatorios demostraron mayores niveles de prejuicio^(27)^. En el presente estudio, las mayores barreras de comunicación se observaron en las subdimensiones de necesidad de atención, curiosidad y búsqueda de información, y tiempo de espera entre los pacientes hospitalizados. Por el contrario, las barreras más bajas se encontraron en las subdimensiones de miedo, ansiedad y prejuicios. Estos hallazgos concuerdan con la literatura, la cual enfatiza que las necesidades de información y atención de los pacientes son prioritarias durante el proceso de tratamiento.

Por ejemplo, Iacorossi et al.^(29)^ evaluaron la calidad de la comunicación entre enfermeras y pacientes entre pacientes oncológicos hospitalizados y destacaron que satisfacer las necesidades de información y atención de los pacientes es fundamental para la satisfacción con la atención. De manera similar, la revisión sistemática de Primeau et al.^(30)^ mostró que los pacientes con cáncer exigen una comunicación empática y clara por parte de los equipos multidisciplinarios al recibir malas noticias. Además de los factores individuales, los determinantes culturales y sistémicos también influyen en la comunicación en la atención oncológica. Por ejemplo, evidencia reciente de América Latina subraya la importancia de abordar las barreras de comunicación en este contexto. Estudios de la región han demostrado que la coordinación limitada dentro de los equipos de salud y el diálogo insuficiente entre el paciente y el proveedor dificultan la comunicación efectiva en la atención oncológica^(31)^. Del mismo modo, se ha observado que los enfoques culturalmente sensibles y basados en la comunidad fortalecen la confianza y el intercambio de información^(32)^. Estos hallazgos concuerdan con nuestro estudio, lo que sugiere que las barreras comunicativas están influenciadas no solo por factores individuales, como el nivel educativo, los síntomas y la presencia de un cuidador, sino también por dinámicas socioculturales y sistémicas más amplias. En este contexto, nuestro estudio realiza una contribución significativa al identificar sistemáticamente las necesidades y barreras comunicativas de los pacientes y sus familiares en el entorno de hospitalización oncológica a nivel subdimensional, subsanando así una laguna en la literatura. En particular, la importancia que se le da a la necesidad de atención y al tiempo de espera resalta la necesidad de intervenciones dirigidas a potenciar la participación activa de los pacientes y sus familiares, así como a mejorar los procesos institucionales en la práctica clínica. Además, la literatura destaca la importancia de una comunicación e interacción efectivas, así como la provisión de protocolos y pautas de atención de seguimiento. Se recomienda que se integren en la práctica clínica más investigaciones sobre la comunicación centrada en el paciente^(10,33)^. Este estudio examina en detalle las barreras de comunicación que enfrentan los pacientes y sus familiares en el contexto de la atención oncológica hospitalaria, lo que permite llenar un vacío en la literatura y brindar orientación para la práctica clínica.

Este estudio encontró que las personas con educación universitaria y superior tenían más curiosidad y necesidades de información que aquellas con educación secundaria y primaria. La información es la necesidad más crítica para los pacientes con cáncer y puede generar barreras para una comunicación eficaz en materia de cuidados de salud^(34,35)^. Las personas con enfermedades que ponen en riesgo la vida, como el cáncer, desean conocer su pronóstico, los tratamientos y los efectos secundarios^(36)^. Al igual que en nuestro estudio, las investigaciones muestran que la necesidad de información sobre la enfermedad aumenta a medida que se elevan los niveles de educación^(37,38)^. Las personas con niveles más altos de educación tienden a tener mayores niveles de alfabetización en salud y una mejor toma de decisiones médicas^(39)^, lo que les permite comunicarse de manera efectiva con los profesionales de la salud^(40)^. Satisfacer las necesidades de información de los pacientes es fundamental para una comunicación efectiva. La información puede reducir la ansiedad y la angustia, apoyar la toma de decisiones y la satisfacción del paciente, y mejorar la capacidad de afrontamiento. Los pacientes con cáncer avanzado suelen recurrir a fuentes de información para comprender la enfermedad y mantener la esperanza^(41,42)^. Evidencia reciente basada en la población de los Estados Unidos también muestra que los niveles más altos de comunicación centrada en el paciente se asocian con menos dificultades y menos frustración durante la búsqueda de información entre los sobrevivientes de cáncer, lo que resalta que tanto la disponibilidad de información precisa como la calidad de la comunicación influyen en las experiencias de los pacientes al lidiar con su enfermedad^(43)^. Por lo tanto, es esencial informar a los pacientes sobre los aspectos que necesitan conocer en todos los procesos, incluyendo el diagnóstico, el tratamiento y el seguimiento.

Este estudio encontró que la necesidad de atención aumentaba con niveles más altos de educación. Los pacientes con cáncer requieren atención para garantizar el control de los síntomas y satisfacer sus necesidades psicosociales y económicas^(44)^. Las expectativas de los pacientes y sus familiares respecto a los profesionales de la salud, independientemente de que se cumplan o no, tienen un impacto en los pacientes y sus familiares. Una atención limitada a las necesidades de las personas con cáncer puede causar barreras de comunicación^(45)^. Una atención inadecuada a las necesidades de los pacientes puede provocar angustia innecesaria, un aumento de los costos de atención médica, una disminución de la calidad de vida y la insatisfacción del paciente^(46)^. Se cree que la necesidad de atención aumenta con el nivel educativo, ya que los pacientes con mayor nivel educativo buscan y siguen fuentes correctas de información sobre el curso de la enfermedad y sus resultados, lo que aumenta su conciencia sobre los posibles síntomas. Para una comunicación saludable, la expectativa de los pacientes de recibir atención por parte del personal de salud se considera un factor positivo.

Sorprendentemente, este estudio encontró que la necesidad de atención disminuía a medida que aumentaba la gravedad de los síntomas. Los síntomas de los pacientes con cáncer avanzado suelen ser numerosos, simultáneos y graves. A medida que aumentan los síntomas que experimentan los pacientes, su estado funcional se deteriora y disminuye su capacidad para realizar actividades cotidianas y socializar^(47)^. En apoyo de esto, un estudio reciente realizado en Turquía reveló que los pacientes con mayor carga de síntomas reportaron niveles más bajos de comunicación centrada en el paciente, lo que resalta el impacto del malestar físico y psicológico en la participación en la comunicación^(28)^. Treiman et al.^(48)^ informaron que los pacientes con cáncer colorrectal que presentaban mala salud física obtuvieron puntuaciones promedio más bajas en la comunicación centrada en el paciente y sus subdimensiones. De manera similar, se observó que los pacientes con un estado de salud deficiente obtuvieron puntuaciones promedio más bajas en la comunicación centrada en el paciente que aquellos con buen estado de salud^(49)^. Si bien los pacientes con cáncer y mal estado de salud necesitan una mayor comunicación centrada en el paciente, las barreras relacionadas con la enfermedad pueden obstaculizar la interacción. Por lo tanto, dar prioridad a los pacientes con peor estado de salud en las intervenciones que abordan las necesidades de supervivencia puede ser esencial para una comunicación centrada en el paciente eficaz^(42)^. Los profesionales de la salud pueden suponer que los pacientes con un estado físico deficiente no pueden participar en la comunicación, lo que puede afectar significativamente la interacción^(49)^. Los hallazgos de este estudio sugieren que los pacientes que experimentan complicaciones intensas, como fatiga, náuseas y dolor derivados del tratamiento contra el cáncer, pueden mostrarse reacios a comunicarse y preferir descansar. Por lo tanto, es esencial reconocer los síntomas graves y no ignorar las necesidades de los pacientes. Se recomienda dar prioridad a los pacientes con mala salud física y mental en la comunicación centrada en el paciente.

Enfrentar una enfermedad que pone en riesgo la vida puede desencadenar reacciones involuntarias como ansiedad, inquietud, desesperanza, enojo, miedo, depresión e impotencia^(47)^. El riesgo de experimentar ansiedad es mayor cuando no hay una persona con quien conviva o cuando el apoyo social es insuficiente^(50)^. Teniendo en cuenta la carga de la enfermedad, especialmente en pacientes con cáncer, este es un tema crítico que debe destacarse^(51)^. Los familiares o acompañantes también pueden desempeñar un papel facilitador en la comunicación al ayudar a los pacientes a obtener y comprender información sobre el proceso de la enfermedad^(10)^. En este estudio, se observó que el miedo y la ansiedad de los pacientes aumentaban en ausencia de un cuidador. Contar con un cuidador es esencial para los pacientes con cáncer a fin de recibir información, acceder a recursos, gestionar el tratamiento y tomar decisiones informadas. Se cree que la ausencia de una persona de apoyo durante la atención, los exámenes, el tratamiento y la hospitalización puede hacer que los pacientes teman no poder manejar adecuadamente el proceso del cáncer y el tratamiento. Por lo tanto, las enfermeras deben apoyar activamente a los pacientes en ausencia de cuidadores para reducir sus miedos y ansiedades. Brindar orientación y estrategias para afrontar los desafíos emocionales y físicos del cáncer es crucial para ayudar a los pacientes a reducir la ansiedad y mejorar el apoyo a quienes se encuentran solos. Investigaciones recientes destacan que las fallas en la comunicación centrada en el paciente pueden exacerbar estos temores, especialmente cuando los sobrevivientes de cáncer sienten que sus necesidades informativas y emocionales no están satisfechas^(52)^.

## CONCLUSIÓN

Una mayor comunicación entre enfermeras y pacientes tendrá implicaciones importantes para los resultados y la calidad de la atención. La incertidumbre puede ser una fuente de estrés y ansiedad para los pacientes y puede afectar su capacidad para tomar decisiones informadas sobre su atención. Las enfermeras deben ayudar a los pacientes a lidiar con la incertidumbre, identificar situaciones que puedan crear barreras de comunicación durante el curso de la enfermedad, satisfacer la necesidad de información y atención, y tomar en cuenta los niveles educativos de los pacientes al brindar todo esto. Dado que la necesidad de atención de las personas con síntomas relacionados con el cáncer puede aumentar, se deben evaluar los síntomas de los pacientes durante la atención. En el caso de síntomas graves que puedan estar relacionados con el cáncer y su tratamiento, no se debe ignorar la necesidad de comunicación del paciente. Se deben identificar los temores y preocupaciones de los pacientes que están solos, es decir, aquellos que no tienen a nadie que los cuide, y se deben atender sus necesidades.

Este estudio debe interpretarse a la luz de ciertas limitaciones. Su diseño transversal impide realizar inferencias causales, y la muestra de un solo centro puede limitar la generalización de los hallazgos. Además, aunque se han corregido las inconsistencias numéricas, no se puede excluir por completo el sesgo de notificación. Por último, el uso de datos autoinformados puede introducir sesgos de recuerdo o de deseabilidad social. A pesar de estas limitaciones, este estudio ofrece información valiosa sobre las barreras de comunicación centradas en el paciente entre los pacientes oncológicos y destaca áreas clave para la mejora clínica. Estos hallazgos también subrayan la importancia de la capacitación específica en comunicación y de las intervenciones sensibles al nivel de alfabetización para el personal de enfermería, lo que sugiere que futuros estudios longitudinales podrían explorar más a fondo las relaciones causales entre las características de los pacientes y las barreras de comunicación en la atención oncológica.

## Data Availability

El conjunto de datos que respaldan las conclusiones de este estudio está disponible previa solicitud al autor correspondiente.
